# New chemically induced skin tumour susceptibility loci identified in a mouse backcross between FVB and dominant resistant PWK

**DOI:** 10.1186/1471-2156-8-39

**Published:** 2007-06-28

**Authors:** Kyoko Fujiwara, Jun Igarashi, Natsumi Irahara, Makoto Kimura, Hiroki Nagase

**Affiliations:** 1 Department of Cancer Genetics, Roswell Park Cancer Institute, Elm and Carlton Streets, Buffalo, New York 14263, USA; 2 Life Science, Advanced Research Institute for the Sciences and Humanities, Nihon University, 6th Ichgaya-Tokyu Building, 4-2-1 Kudan-kita, Chiyoda-ku, Tokyo, 102-0073, Japan; 3 Division of Cancer Genetics, Department of Advanced Medical Research, Nihon University School of Medicine, 30-1 Oyaguchi, Kami-cho, Itabashi-ku, Tokyo, 173-861, Japan

## Abstract

**Background:**

A variety of skin cancer susceptibility among mouse strains has allowed identification of genes responsible for skin cancer development. Fifteen *Skts *loci for skin tumour susceptibility have been mapped so far by using the two-stage skin carcinogenesis model [induced by 7.12-dimethylbenz(a)anthracene (DMBA)/12-O-tetradecanoylphorbol-13-acetate (TPA)]. A few responsible genes have been identified using wild-derived dominant resistant *Mus spretus *mice, and one has been confirmed as a low penetrance cancer susceptibility gene in a variety of human cancers.

**Results:**

In the present study, we found that wild-derived PWK mice developed no tumour by treatment with the two-stage skin carcinogenesis protocol. This phenotype is dominant resistant when crossed with the highly susceptible strain FVB. By analyzing the F1 backcross generation between PWK and FVB, we found empirical evidence of significant linkage at the new loci *Skts-fp1 *on chromosome 4 and suggestive linkage on chromosomes 1, 3, 11, 12 and 14 for skin tumour susceptibility. *Skts-fp1 *includes the *Skts7 *interval, which was previously mapped by a *Mus spretus *and NIH backcross. We also observed suggestive linkage on chromosomes 1 and 2 in the female population only, while suggestive linkage on chromosomes 14 and 15 only was observed in the male population. A significant genetic interaction was seen between markers of *D11Mit339* and *D16Mit14*.

**Conclusion:**

Analysis of this new cross may facilitate the identification of genes responsible for mouse skin cancer susceptibility and may reveal their biological interactions.

## Background

Germ-line mutation has been reported in tumour suppressor genes and in oncogenes that are responsible for the highly penetrated familial cancer syndrome [1]. The majority of cancers, however, are sporadic and often associated with familial aggregation. Therefore, cancer susceptibility in general is explained by multiple low penetrance cancer susceptibility genes [2,3].

Although analysis of the human population is useful in finding the gene related to high penetrance disease, the identification of low penetrance tumour susceptible genes is still difficult. This is mainly due to heterogeneous genetic background and complex environmental effects. Mouse models of cancer have been used extensively for the analysis and identification of the genetic components of tumour susceptibility [4,5]. The susceptibility for the two-stage skin carcinogenesis model varies among mouse strains [6,7], and the genetic approach has been performed to identify genes related to cancer susceptibility [8-11]. Previous studies indicate that the percent contribution of skin cancer susceptibility can be explained by each genetic component consisting of one of multiple loci and their interaction [12]. Using NIH and *Mus spretus *crosses, in which *Mus spretus *is dominant resistant against susceptible *Mus musculus *strains, skin cancer susceptibility loci *Skts1~15 *were mapped [8,9,12]. *Psl1~4 *were mapped by using C57BL/6J(B6) and DBA crosses, in which DBA shows semi-dominant susceptibility against resistant B6 [10,13]. Tomaso Dragani's group also mapped *Skts1 *using Car-R and Car-S outbred. crosses [14], which were generated by balanced intercross of inbred strains, and reported loss of tyrosinase activity conferred increased skin tumor susceptibility [15]. *Skts13 *was identified as the Aurka gene [16], and *Skts14 *was identified as *Tgfb1 *[11]. However, none of the responsible genes have been cloned yet at the remaining loci. Since functional polymorphisms of *AURKA *were repeatedly reported to be determinants of individual risk for colon, esophageal, skin, lung, breast, ovarian, and prostate cancers in the human population [16-19], identification of genetic components in the mouse skin cancer susceptibility could facilitate the identification of candidate cancer risk factors in the human population.

To narrow down the candidate loci and to identify the corresponding gene for the genetic components of mouse skin cancer susceptibility, information from other strains and crosses are valuable. In this study, we found that the PWK strain, which is an inbred mouse derived from wild *Mus musculus musculus *mice [20], is dominantly resistant to chemically induced skin carcinogenesis when crossed with a highly susceptible FVB strain. Because of the dominant resistance and high genetic diversity between PWK and FVB strains [21], we expected that there were multiple genetic components involved in skin cancer susceptibility as seen in our previous report of NIH/*spretus *cross. Therefore, we performed genetic analysis of a large backcross between PWK and FVB strains.

## Results

Using the two stage skin tumour induction treatment, all 16 FVB mice developed papillomas with an average multiplicity of 32.56 ± 3.6, at 20 weeks after the initiation. However, none of the 10 PWK/Rbrc mice developed any papillomas on dorsal skin (Table 1). Also, none of the 17 (PWK/Rbrc × FVB/N)F1 mice, abbreviated as PF, developed tumours. Four out of the 25 (FVB/N × PWK/Rbrc)F1 mice, abbreviated as FP, developed tumours, but the average multiplicity was 0.32 ± 0.16 (Table 1). These results demonstrate that the PWK strain has a dominant resistant phenotype to the DMBA/TPA induced skin tumour against a susceptible FVB strain.

**Table 1:** Incidence and multiplicity of papilloma in the parental strains, F1 and F1 backcross

Strain		Animal No.	Incidence (%)	Multiplicity ^a)^
FVB/N	total	16	16 (100)	32.56 ± 3.60
	female	9	9 (100)	28.22 ± 4.46
	male	7	7 (100)	38.14 ± 5.36
PWK/Rbrc	total	10	0 (0)	0
	female	10	0 (0)	0
	male	10	0 (0)	0
FVB × PWK	total	25	4 (16)	0.32 ± 0.8
	female	14	4 (28.6)	0.57 ± 1.01
	male	11	0 (0)	0
PWK × FVB	total	17	0 (0)	0
	female	8	0 (0)	0
	male	9	0 (0)	0
(FxP) × F	total	208	123(56.7)	4.86 ± 0.56
	female	107	60(56.1)	3.45 ± 0.64
	male	101	63(62.4)	6.34 ± 0.92

^a) ^data are shown as mean ± SEM.

**Figure 1:**
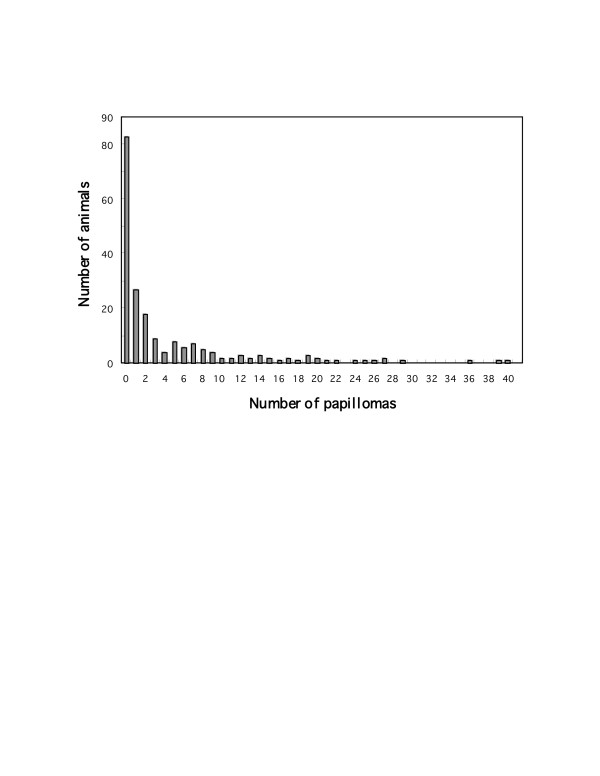
Distribution patterns of papilloma multiplicity. The number of papillomas 20 weeks after the initiation is plotted as histograms.

Linkage analysis was performed by genotyping 208 F1 backcross mice (abbreviated as FPxF) for 204 polymorphic markers spread throughout the genome. The number of papillomas 20 weeks after the initial treatment was used as a trait. One hundred twenty three mice (56.7%) developed papillomas, and average multiplicity was 4.86 ± 0.56 (Table 1, Fig. 1). Males developed a significantly higher number of papillomas than females. Even though it was not significantly associated, FVB males also showed higher multiplicity than FVB females. On the contrary, only female mice developed papillomas in the F1 hybrids (Table 1).

**Figure 2:**
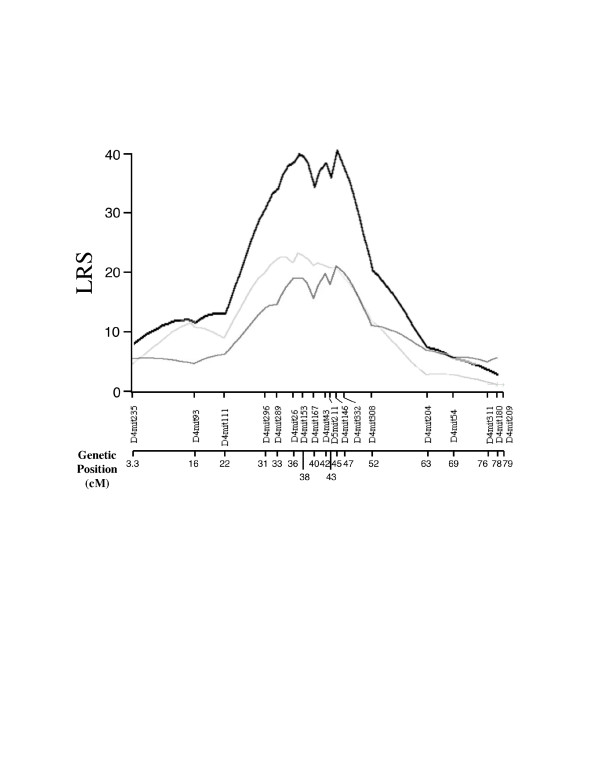
Interval mapping on chromosome 4. The QTL on chromosome 4 shows significant linkage to multiplicity of papilloma 20 weeks after the initiation. Black line indicates the linkage calculated for whole mice, right gray is for female mice, dark gray is for male mice. Genetic distances of each marker from centrosome are indicated as genetic position (cM) on the bottom.

For parametric quantitative trait loci (QTL) regression analysis, tumour multiplicity data are transformed by root-square transformation to improve the fit of a negative binomial distribution pattern of nominal data sets to a normal distribution [22]. By single point marker regression analysis, significant linkage with likelihood ratio statistics (LRS) larger than 12.9 was mapped only on chromosome 4 (Table 2). LRS of at least 6.9 was considered evidence for suggestive linkage and was detected on chromosomes 1, 3, 4, 11, 12 and 14. Interval mapping on chromosome 4 showed highly significant linkage between markers *D4Mit111 *and *D4Mit308 *(*Skts-fp1*). This QTL interval covered almost one third of chromosome 4 and there seem to be at least two peaks close to *D4Mit26 *and *D4Mit146 *with values of LRS 38.5 and 40.5, respectively (Fig. 2). It was estimated that approximately 18% of the total trait variance would be explained by a QTL at this locus.

**Table 2:** QTL for papilloma multiplicity

		total						female		male	
				
				Number of papillomas ^c)^							
								
Marker	Map location ^a)^	LRS	P-value ^b)^	FF (Number of animals)		FP (Number of animals)		LRS	P-value ^b)^	LRS	P-value ^b)^
Chromosome1											
D1Mit18	21.4	7.4	0.00671*	6.33 ± 0.96	(103)	3.37 ± 0.56	(104)	8.9	0.00288*	0.9	>0.01
D1Mit284	35.6	6.8	0.00924					9.4	0.00219*	0.9	>0.01
D1Mit10	44.1	6.0	>0.01					8.4	0.00383*	0.7	>0.01
Chromosome2											
D2Mit484	50	1.7	>0.01					8.1	0.00445*	0.2	>0.01
D2Mit401	58.1	5.2	>0.01					8.9	0.00289*	0.5	>0.01
D2Mit423	63.7	3.2	>0.01					8.8	0.00308*	0.0	>0.01
Chromosome3											
D3mit40	36.1	7.6	0.0058*	6.39 ± 0.94	(109)	3.19 ± 0.54	(98)	3.6	>0.01	2.8	>0.01
Chromosome4											
D4Mit235	3.3	7.4	0.00648*	6.56 ± 0.98	(98)	3.33 ± 0.57	(110)			4.1	>0.01
D4Mit93	16.1	11.0	0.00092*	6.55 ± 0.94	(99)	3.31 ± 0.60	(109)	10.4	0.00123*	3.2	>0.01
D4Mit111	22.1	12.5	0.00041*	6.57 ± 0.94	(99)	3.29 ± 0.61	(109)	8.8	0.00354*	4.8	>0.01
D4Mit26	36.3	34.4	<0.00001***	7.94 ± 1.05	(95)	2.11 ± 0.39	(109)	16.5	0.00005**	18.7	0.00002**
D4Mit167	40	34.2	<0.00001***	7.82 ± 1.01	(100)	2.11 ± 0.39	(108)	21.5	<0.00001***	15.0	0.00011**
D4Mit308	51.9	19.9	0.00001**	6.72 ± 0.86	(100)	3.12 ± 0.69	(108)	11.6	0.00067*	10.1	0.00147*
Chromosome11											
D11Mit339	33.8	8.3	0.00388*	6.36 ± 0.92	(99)	3.48 ± 0.65	(109)	3.3	>0.01	5.3	>0.01
Chromosome12											
D12Mit283	9.4	7.8	0.00534*	6.24 ± 0.92	(107)	3.39 ± 0.60	(101)	2.5	>0.01	4.6	>0.01
D12Mit153	15.1	7.0	0.0081*	6.25 ± 0.93	(105)	3.44 ± 0.60	(102)	3.0	>0.01	3.3	>0.01
Chromosome14											
D14Mit257	24.6							0.6	>0.01	9.4	0.00222*
D14Mit203	44.1	8.4	0.00382*	6.18 ± 0.89	(100)	3.63 ± 0.68	(108)	1.8	>0.01	5.9	>0.01
D14Mit193	48.3	12.3	0.00046*	6.43 ± 0.88	(103)	3.31 ± 0.67	(105)	1.8	>0.01	11.0	0.00089*
D14Mit195	60.3	8.0	0.00465*	5.96 ± 0.78	(107)	3.68 ± 0.81	(101)	1.5	>0.01	6.2	>0.01
D14Mit197	72.7							1.0	>0.01	8.0	0.00475*
Chromosome15											
D15Mit15	64.9	3.4	>0.01					1.7	>0.01	10.6	0.0011*
D15Mit161	68.8	4.8	>0.01					0.2	>0.01	9.1	0.0026*

^a) ^cM from the centrosome

^b) ^P-value for the association at each locus

^c) ^Data are shown as mean ± SEM.

Values in parenthesis are number of samples.

* Suggestive linkage

** Significant linkage

*** Highly Significant linkage

**Figure 3:**
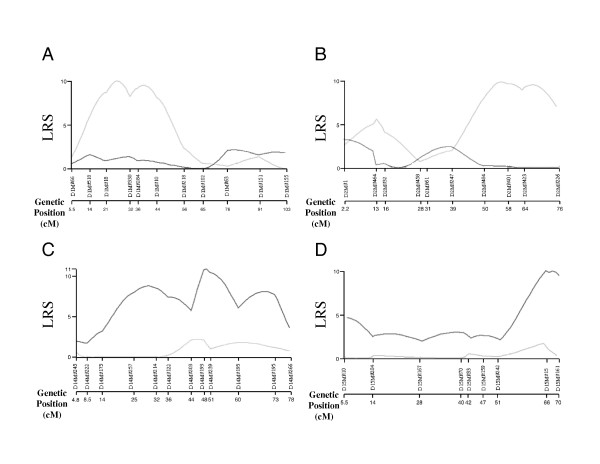
Interval mapping on chromosome 1, 2, 14 and 15. Result of interval mapping on (A) chromosome 1, (B) chromosome 2, (C) chromosome 14 and (D) chromosome 15 are shown. Right gray line indicates the linkage for female mice and dark gray line for male mice. Genetic distances of each marker from centrosome are indicated as the genetic position (cM) on the bottom.

The suggestive linkage on chromosomes 1, 2, 14 and 15 showed gender difference. Linkage on chromosomes 1 and 2 was observed only in female, and linkage on chromosomes 14 and 15 was in males exclusively. Suggestive evidence of linkage at loci on chromosome 1, 2, 14 and 15 was obtained with interval mapping in either male or female population exclusively (Fig. 3A~D).

**Figure 4:**
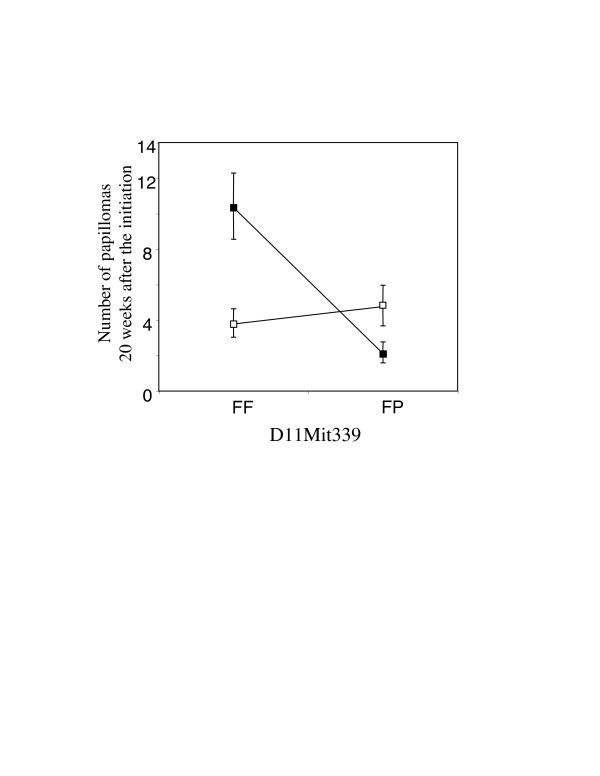
Allelic interaction between *D11Mit339 *and *D16Mit14*. Association between *D11Mit339 *genotype and multiplicity of papilloma 20 weeks after the initiation is shown. Black square indicates the mice with FF genotype at the *D16Mit14 *locus. White square indicates the mice with FP genotype at the *D16Mit14 *locus. Data are shown as mean ± SEM.

**Figure 5:**
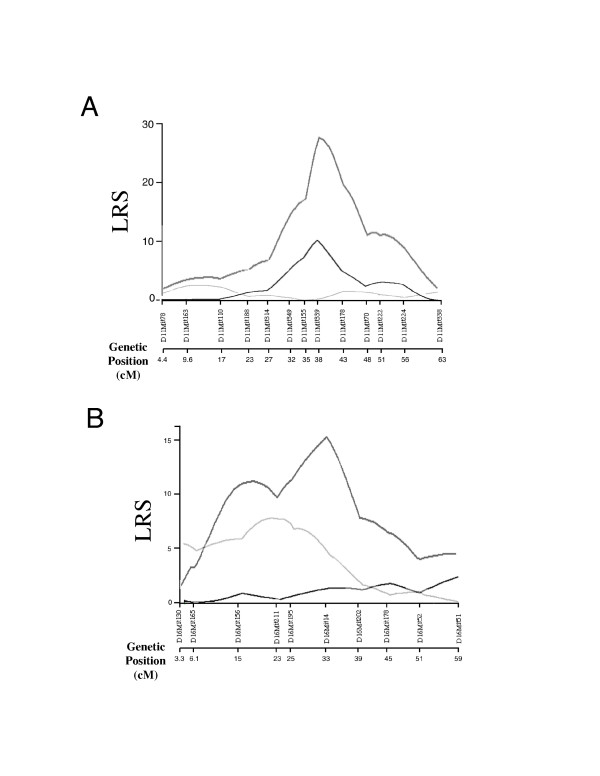
Interval mapping on chromosome 11. (A) Interval mapping results on chromosome 11 are shown for the mice with FF genotype (dark gray line) and the mice with FP genotype (right gray line) at the *D16Mit14 *locus. Black line indicates interval mapping for whole mice. Significant linkage is observed only in the mice with FF genotype at the *D16Mit14 *locus. (B) Interval mapping results on chromosome 16 are shown for the mice with FF genotype (dark gray line) and the mice with FP genotype (right gray line) at the *D11Mit339 *locus. Black line indicates interval mapping for whole mice. Significant linkage is observed only in the mice with FF genotype at the *D11Mit339 *locus. Genetic distances of each marker from centrosome are indicated as the genetic position (cM) on the bottom.

To find interaction components among QTLs, a two-dimensional scan of per-mutation test using J/qtl was performed. With a standard criteria (Joint LOD>5 and [Epistatic LOD > 3 or minimum LOD {locus1, locus2} >3]), possible interactive QTL with epistatic LOD 4.14 was found between markers *D11Mit339 *and *D16Mit14*. A plot of the average number of papillomas of each genotype at *D11Mit339 *and *D16Mit14 *is shown in Fig. 4. Homozygous FVB allele (FF) at the *D16Mit14 *marker is associated with susceptibility if the genotype at the interacting locus *D11Mit339 *is a homozygous (FF) genotype. In the mice with heterozygous FP genotype at *D16Mit14*, genotype at *D11Mit339 *had no association with the multiplicity of papillomas. Interval mapping indicated a highly significant linkage *Skts-fp2 *(LRS 27.6) between markers *D11Mit155 *and *D11Mit178 *locus in mice with FF at *D16Mit14*, but no linkage was observed in mice with FP at *D16Mit14 *(Fig. 5A). By interval mapping, significant linkage *Skts-fp3 *(LRS15.3) was also mapped between markers *D16Mit202 *and *D16Mit195 *in mice with FF genotype at *D11Mit339 *(Fig. 5B). Some weak linkage was observed also in mice with FP at *D11Mit339*, however the additive effect was opposite, i.e. PWK allele on chromosome16 could be susceptible allele for skin tumor development in FP at *D11Mit339*, while it is resistant in FF at *D11Mit339*. Approximately 26% of the total trait variance would be explained by *Skts-fp2 *in mice with FF genotype at *D16Mit14 *locus, and 14% for *Skts-fp3 *in mice with FF genotype at *D11Mit339*. No sexual difference was observed in *Skts-fp2 *and *Skts-fp3*.

## Discussion

The present study indicates that PWK is a dominant resistant strain to two-stage skin carcinogenesis, when crossed with highly susceptible FVB mice. Among the 20 mice in each group, no PWK developed papillomas, and only a few FP F1 hybrid mice developed one papilloma. In a 208 backcross mice study, which should detect QTLs with more than 4% variance, a significant QTL of *Skts-fp1 *has been mapped. The linkage study using genetically and phenotypically diverged strains FVB and PWK can facilitate analysis and understanding the genetic component of this complex cancer susceptibility.

PWK is a wild-derived strain that could help ensure high genetic diversity [21,23]. As seen in NIH and *Mus spretus *inter-specific crosses, we expected to have a large genetic variation to skin tumour susceptibility between FVB and PWK. However, we mapped a significant locus on chromosome 4 only. Interval mapping indicated that *Skts-fp1 *showed multiple significant peaks located between *D4Mit111 *and *D4Mit308*, which covered almost one third of chromosome 4, suggesting that a linkage group consisting of multiple genes in a chromosome may be involved in *Skts-fp1*. Contributions of the *D4Mit26 *locus and the *D4Mit146 *locus (two major linkage peaks between which recombination are observed in 16 mice) are estimated to explain the approximately 15% and 18% of the variance of PWK resistant phenotype, respectively. These contributions are higher than the contribution of major QTLs in the NIH and *Mus spretus *backcross study, indicating that contributions on a single chromosome are important in the FVB and PWK cross. Linkage analysis also showed suggestive linkage at loci on chromosomes 1, 3, 11, 12 and 14 in addition to significant linkage at *D4Mit26 *and *D4Mit167 *(*Skts-fp1*). Although additional markers and backcross mice are needed to hypothesize the multiple genetic effects in this cross, it is possible that many other weak loci and epistatic components contribute to the resistant phenotype of PWK.

*Skts7*, which was mapped as a QTL on chromosome 4 for chemically induced skin tumour susceptibility by the analysis of the NIH/Ola and *Mus spretus *cross [8,9], is located within *Skts-fp1*. A common gene may be responsible for the skin susceptibilities of the two crosses, segregating FVB with PWK and NIH with *Mus spretus*. So far, the SNP information in the Phenome database is not sufficient to find common haplotype blocks between PWK and *Mus spretus*. However, after narrowing down the locus and carrying out expression and functional analysis of the candidate genes, we can perform a haplotype analysis identifying a common polymorphism in the gene between resistant strains (*Mus spretus*, PWK) and susceptible strains (NIH, and FVB). The haplotype analysis will help to identify a candidate skin resistant gene.

From the study with Ornithine decarboxylase (Odc) transgenic BALB/cJ and C57BL/6J mice, significant linkage of the skin tumour induced by DMBA and the Odc transgene was detected in the interval between *D4Mit31*and *D4Mit52 *(51.3–54.9 cM) [24]. These two strains may also share a common resistant allele and could be used for haplotype mapping to identify the candidate.

On this interval of *Skts-fp1*, many QTLs for susceptibility to other types of tumours have been mapped [4,5]. *Pctr1 *and *Pctr2 *have been mapped as loci for susceptibility to pristine induced plasmacytoma [25,26], and *Cdkn2a *has been suggested to be a corresponding gene for *Pctr1*[27,28]. *Cdkn2a *is also a strong candidate for *Papg1*, QTL for progression of urethane induced lung tumours [29]. The *Cdkn2a *locus encodes two tumour suppressors, P16^INK4a ^and P19^ARF^. There are no non-synonymous polymorphisms between FVB and PWK in ankirin 1 and 2 domains of P16^INK4a^, which are suggested to be corresponding polymorphisms for *Pctr1 *and *Papg1 *(data not shown). Because Figure 2 shows multiple peaks at the *Skts-fp1 *locus on chromosome 4, as seen with other tumour susceptibility QTLs [30], the locus is complex and may create a linkage group containing two or more overlapping susceptibility and/or resistant regions. Thus, we have initiated the creation of congenic mice containing segments of the donor PWK allele on chromosome 4 to the recipient FVB background.

*Skts-fp2 *on chromosome 11 was identified as a significant linkage among FVB homozygous mice at marker *D16Mit14*, but not in mice heterozygous at this locus. *Skts-fp3 *on chromosome 16 was also identified among FVB homozygous mice at the *D11Mit339 *locus, but not in mice with heterozygous at this locus. Our results suggest that *Skts-fp2 *and *Skts-fp3 *showed the evidence of interaction. An interaction component between two loci has been suggested to play an important role in cancer susceptibility [4,12] and recently *Tgfb1 *was indicated as a corresponding gene for a skin tumour susceptible locus *Skts14*. This locus was shown to have interaction with *Skts15*, which overlaps with the *Tgfbm3 *locus mapped as *Tgfb1 *modifier locus [11]. *Skts-fp2 *could also be functionally related to *Skts-fp3*. *Scc15*, the QTL for 1,2-dimethylhydrazine induced colon tumour susceptibility in mice [31] and *Sluc4*, the QTL for N-ethyl-N-nitrosourea induced lung tumour in mice [32] have been mapped around the *Skts-fp2 *locus, but no QTL for skin tumor susceptibility is mapped at this locus. No QTL for susceptibility to skin cancer or other types of tumour has been mapped around the *Skts-fp3 *locus, however *Skts9 *for DMBA/TPA induced skin cancer [9] and *Sluc27 *for N-ethyl-N-nitrosourea induced lung tumour [33] are mapped on the same chromosome. If extreme phenotypic differences exist between two strains and dominant interactions are expected, the interaction components can be detected more efficiently using a simple backcross [12]. Although further analysis with an additional number of mice is needed to identify responsible genes at the interactedlocus and significant interaction component between two QTLs, the genetic interaction between *Skts-fp2 *and *Skts-fp3 *reveals a biological interaction of genes at two loci.

In this study, we found that linkages at four loci show sexual difference. Suggestive linkage on chromosomes 1 and 2 was exclusively observed in females and suggestive linkage on chromosomes 14 and 15 was observed only in males. Usually this kind of study is performed with either all female or all male animals, and sexual differences in tumour susceptibility genes are not identified. An increased number of mice will enable us to reveal the QTL for tumour susceptibility, which interacts with sex chromosomes directly or indirectly.

The absence of PWK alleles at both markers remarkably increased the average number of papillomas, whereas no effect was seen in animals that retained one PWK allele at either locus. A dominant resistant to cancer development may be redundant regulation. Candidate accessory regulatory genes modify a major effect gene and can be identified as a dominant interacted modifier. Although individual modifying genes may show only subtle quantitative effects on tumourigenesis, combinations of suppressive alleles can completely eliminate neoplasia. The identification of effective combinations of gene activities reflects the general finding of "genetic redundancy", in which the loss of function of a single gene has no obvious phenotype.

Here we present three significant loci related to skin tumour susceptibility from the analysis of a new strain combination of FVB and PWK. Among previous studies using other tumour induction systems, many tumour susceptible loci mapped on the same chromosomal regions where skin cancer susceptibility loci were mapped. For example, *Psl2 *was mapped close to *Skts13*, *Psl3 *was mapped close to *Skts8*, and the locus found from the analysis of Car-S and Car-R strains was located on the *Skts1 *locus. Also, *Skts-fp1 *identified in this study covered the region on which *Skts7 *and the linkage obtained from ODC transgenic mice have been mapped. As described in the studies for *in silico *mapping methods using many inbred strains, congenic, recombinant inbred, advanced intercross, chromosome substitution strains or very recently collaborative crosses [34-36], it is obvious that accumulated information from several genetic analyses using many other strains provides an important and rapid route to identification of complex germ-line genetic variants that confer increased cancer risk.

## Conclusion

This study shows that PWK is a dominant resistant strain to two-stage skin carcinogenesis when it is crossed with the FVB strain. By linkage analysis using backcross between FVB and PWK, three significant QTLs, *Skts-fp1, Skts-fp2 *and *Skts-fp3*, for the skin tumour susceptibility locus were mapped. Studies using this new cross could be an efficient method for finding new genes responsible for skin cancer susceptibility and for examining genetic/biological interaction between the responsible genes.

## Methods

### Animals

Mice used in this study were bred in the SPF Facility in the Department of Laboratory Animals at Roswell Park Cancer Institute and are treated in accordance with IACUC regulations. FVB/N mice were purchased from TACONIC (Germantown, NY). PWK/Rbrc inbred mice were obtained from RIKEN BRC (Ibaraki, Japan). Twenty five (FVB/N × PWK/Rbrc) F1 and 17 (PWK/Rbrc × FVB/N) F1, abbreviated as FP and PF respectively, were generated from the two strains of mice. FP mice were then backcrossed with FVB/N to generate 208 F1 backcross animals.

### Skin cancer induction

7.12-Dimethylbenz(a)anthracene (DMBA) and 12-O-tetradecanoylphorbol-13-acetate (TPA) were purchased from Sigma Chemical Co. (St. Louis, MO). DMBA is used as a carcinogen and TPA as a promoter.

Nine of FVB female and 7 of male mice, each 10 female and 10 male of PWK mice, 14 female and 11 male of FP hybrids, 8 female and 9 male of PF mice, 107 female and 101 male of FPxF backcross mice were applied for skin carcinogen.

At 8–11 weeks of age, the back skin of each mouse was carefully shaved with an electric clipper. Two days after shaving, 200 ul of DMBA (0.125 mg/ml) was dissolved in acetone and applied to the back of each mouse prior to the hair growth cycle. A total of 97.4 nmol of DMBA was applied to each mouse. One week after the treatment, mice received 400 ul of TPA (5 × 10^-5 ^M solution in acetone) as a promoter, i.e., 32.4 nmol of TPA, twice weekly for 20 weeks. Animals are assessed twice weekly for the appearance of papillomas during the promotion phase. The number of papillomas was counted every other week until 20 weeks after tumour initiation. Papilloma incidence during the promotion phase and papilloma multiplicity 20 weeks after the initiation was used for assessment of skin tumour susceptibility in this study.

### Genotype

Genomic DNA was isolated from mouse tails using the alkali method. Briefly, 1~2 mm of tails were boiled in 300 ul of 50 mM NaOH for 30 min, then 25 ul of 1 M Tris-HCL (ph8.0) was added. DNAs were then genotyped for 204 polymorphic microsatellite markers spaced at approximately 8-cM intervals through chromosome 1 to 19 and X by polymerase chain reaction (PCR). Information of MIT markers used in this study is available upon request. PCR reactions were carried out using a Biometlar thermocyler, and PCR products were separated by electrophoresis through 4% Nusieve-GTG low melting temperature agarose gels (FMC) in the 0.5 × TBE buffer.

### Statistical analysis

MapManager QTX ver1.03 [37,38] was used to analyse phenotype and genotype data to map QTLs related to susceptibility for chemically induced skin tumours. Permutation tests were performed for FPxF F1 backcross generation, to estimate empirical threshold values for quantitative trait mapping. Single-point marker regression was performed for the whole chromosomes to find linkage, and then interval mapping was performed to analyse the chromosome with significant linkage in the single point marker regression. A permutation test (10,000 permutations at a 1 cM interval) was performed, and the significance threshold was calculated to establish the empirical significance levels in QTL mapping experiments. Suggestive (LRS > 6.9), significant (LRS > 12.9), highly significant (LRS > 20.8) thresholds were established and used for present analysis. A two-dimensional genome scan using J/qtl [39,40] was performed to analyse interactions between QTLs.

## Abbreviations

DMBA: 7.12-dimethylbenz(a)anthracene, TPA: 12-O-tetradecanoylphorbol-13-acetate, F: FVB/N, P: PWK/Rbrc, FP: (FVB/N × PWK/Rbrc)F1 mice, PF: (PWK/Rbrc × FVB/N)F1 mice, FPxF: (FVB/N × PWK/Rbrc) × FVB/N F1 backcross mice, LRS: likelihood ratio statistics, B6: C57BL/6J, Odc: Ornithine decarboxylase, PCR: polymerase chain reaction

## Authors' contributions

JI and KF carried out skin carcinogen experiments. KF analysed the data of skin tumour developed in mice. KF and NI performed genotyping of F1 backcross mice and linkage analysis. HN (corresponding author) is the PI of the program that funded the work and designed this study. This manuscript was written by KF and HN. All authors read and approved the final manuscript.
